# Serum CCL2 and CCL7 in Prostate Cancer: Evaluation of Their Diagnostic Utility in Comparison with Benign Prostatic Hyperplasia and Healthy Controls

**DOI:** 10.3390/ijms27146197

**Published:** 2026-07-11

**Authors:** Weronika Sokólska, Monika Zajkowska, Adam Rafał Nowiński, Karolina Orywal

**Affiliations:** 1 Department of Biochemical Diagnostics, Medical University of Bialystok, Waszyngtona 15A, 15-269 Bialystok, Poland; weronika.sokolska@sd.umb.edu.pl; 2 Department of Neurodegeneration Diagnostics, Medical University of Bialystok, Waszyngtona 15A, 15-269 Bialystok, Poland; monika.zajkowska@umb.edu.pl; 3 Department of Urology, Independent Public Health Care Center of the Ministry of the Interior and Administration in Białystok, Fabryczna 27, 15-471 Białystok, Poland

**Keywords:** prostate cancer, chemokines, CCL2, CCL7, serum biomarkers, cancer diagnostics, immune signaling, tumor microenvironment

## Abstract

Prostate cancer (PCa) is the most common malignancy in men. Recent evidence indicates a correlation between modulation of the tumor microenvironment, tumor growth and metastasis. Chemokines, including C-C chemokine ligand 2 (CCL2) and C-C chemokine ligand 7 (CCL7), participate in regulating inflammation, angiogenesis, and the recruitment of immunosuppressive cells. This study aimed to assess the diagnostic potential of serum CCL2 and CCL7 in patients with PCa, benign prostatic hyperplasia (BPH) and healthy controls. The study included 53 patients with PCa, divided into groups at low, intermediate, and high risk of disease progression, 55 with BPH, and 30 healthy controls. CCL2 and CCL7 concentrations were measured using a multiplex Luminex assay. Serum CCL7 concentration in PCa patients was significantly lower compared to controls. No significant differences in its concentration were found between PCa and BPH patients or between PCa subgroups at different risk levels. CCL7 demonstrated promising sensitivity (96.2%) and AUC (0.917). However, its ability to distinguish PCa from BPH was limited. CCL2 concentrations were significantly lower in the PCa group only compared to the BPH group. These results suggest that CCL7 may represent a potential adjunct marker within multimarker diagnostic strategies for prostate cancer, while CCL2 showed limited diagnostic utility.

## 1. Introduction

Prostate cancer (PCa) is the most common malignancy in men and the second leading cause of cancer death worldwide after lung cancer [1]. It most commonly affects men aged 45 to 60 [2]. The only established risk factors include advanced age, African descent, and a family history of the disease [3]. Genetic mutations and hereditary susceptibility are key determinants of prostate cancer pathogenesis. Genes that increase PCa susceptibility include *HOXB13*, *HPC*, *PCAP*, *HPCX*, *CAPB*, *HPC20*, *MSH2*, and *MSH6* [4]. Furthermore, studies have confirmed the significance of *BRCA1/2* mutations, originally associated with breast and ovarian cancer [5]. One of the biological processes underlying PCa carcinogenesis is androgen signaling, mediated by the androgen receptor (AR) [6]. Disturbance of cell cycle regulation through inactivation of tumor suppressors, including the fundamental role of PTEN phosphatase, translates into constitutive AKT activation, NF-κB-driven stemness, and evasion of growth suppression. The interplay between the PTEN/PI3K/AKT pathways, p53 protein dysfunction, and additional overexpression of the MYC proto-oncogene impairs the response to DNA damage, promoting genomic instability and driving the proliferation of mutant cells [7].

Evasion of the immune system is another process contributing to prostate cancer progression. Immunosuppression, characterized by reduced cytotoxic T cell activity, impaired antigen presentation, and elevated levels of immunosuppressive cytokines and immune checkpoint molecules, combined with the concomitant infiltration of regulatory T cells (Tregs) and tumor-associated macrophages (TAMs), contributes to a poorer prognosis. Recent evidence indicates a strong correlation between modulation of the tumor immune microenvironment (TIME) and tumor growth and metastasis [8], and chemokine signaling is an important element of information exchange and a specific link between ongoing biological processes, either enhancing or muting their effects [9].

The C-C motif chemokine ligand 2 (CCL2) and the C-C motif chemokine ligand 7 (CCL7) are proinflammatory proteins belonging to the CC chemokine family. They recruit monocytes and other CCR2-expressing immune cells to the tumor microenvironment (TME). Beyond their chemotactic activity, both chemokines shape the composition and functional state of the TME by promoting the recruitment and polarization of tumor-associated macrophages and other immunosuppressive myeloid cells, thereby facilitating immune evasion and tumor progression [10,11].

**Figure 1:**
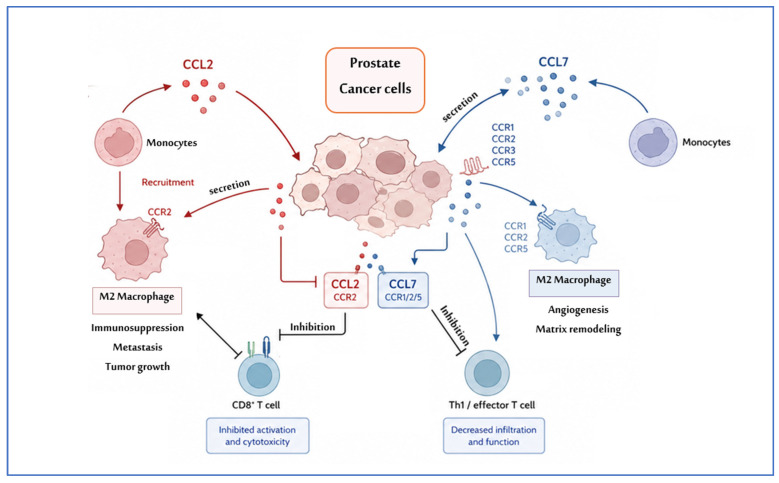
The role of chemokines CCL2 and CCL7 in the prostate cancer environment.

Both chemokines are ligands of the CCR2 receptor, which allows for functional redundancy whereby multiple ligands can exert similar biological effects through a single receptor [12]. Numerous studies suggest their role in tumorigenesis by promoting angiogenesis and stimulating the influx of immunosuppressive myeloid cells, including TAMs, and myeloid stem cell-derived suppressor cells (Figure 1) [13,14]. Although several studies have investigated the expression of CCL2 and CCL7 in prostate cancer tissues, data regarding circulating levels of these chemokines and their diagnostic value remain limited and inconclusive. In particular, there is a paucity of studies evaluating serum CCL7 concentrations in the context of prostate cancer risk and diagnosis. Therefore, further research is warranted to clarify the potential clinical utility of these chemokines as non-invasive biomarkers.

Currently, total prostate-specific antigen (tPSA) remains the most widely used biomarker in prostate cancer diagnostics. However, despite its established clinical value, tPSA has several limitations, particularly regarding specificity and its ability to distinguish malignant from benign prostate conditions [15].

However, data on the diagnostic value of circulating CCL7 in prostate cancer remain limited. Although CCL2 has been extensively studied in the context of prostate cancer, considerably less is known about the clinical significance of circulating CCL7. Furthermore, to the best of our knowledge, no studies have directly compared the diagnostic utility of both chemokines within the same patient cohort comprising individuals with prostate cancer, benign prostatic hyperplasia, and healthy controls. Given the current state of knowledge and emerging evidence indicating an important role of CCL2 and CCL7 in cancer development and progression, we conducted this study to evaluate these chemokines as novel serum biomarkers and their diagnostic utility in patients with prostate cancer. We hypothesized that circulating levels of CCL2 and CCL7 differ between prostate cancer, benign prostatic hyperplasia, and healthy individuals and may provide additional diagnostic information beyond PSA. Therefore, this study was designed to address this knowledge gap and to determine whether CCL7 provides complementary diagnostic information beyond the better-characterized CCL2.

## 2. Results

The concentrations of chemokines CCL2 and CCL7 measured in the serum of patients with prostate cancer (PCa), patients with benign prostatic hyperplasia (BPH), and healthy volunteers (control group) are summarized in Table 1.

**Figure 2:**
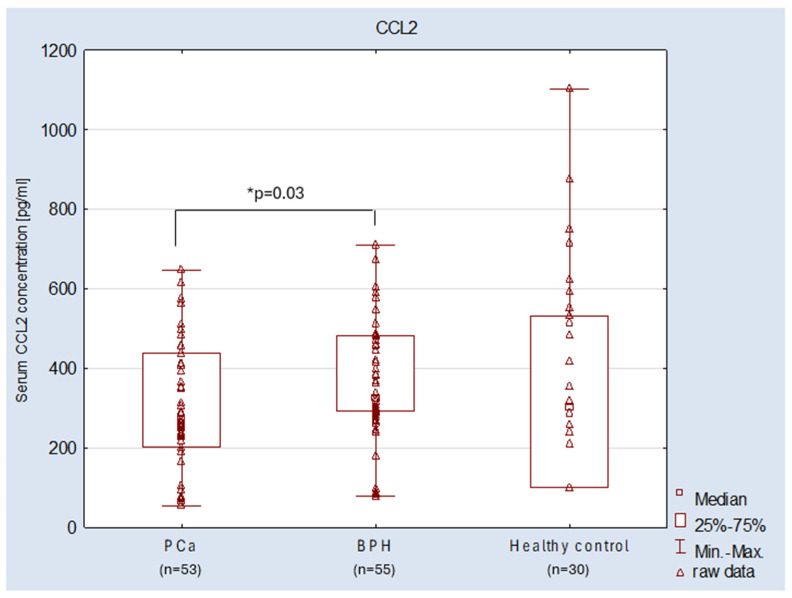
Serum concentrations of CCL2 in patients with prostate cancer (PCa), benign prostatic hyperplasia (BPH), and healthy controls. The box plots illustrate the median (central line), interquartile range (IQR; box), and minimum–maximum range (whiskers), while individual data points indicate single observations. Statistical analysis was performed using the Mann–Whitney U test.

**Figure 3:**
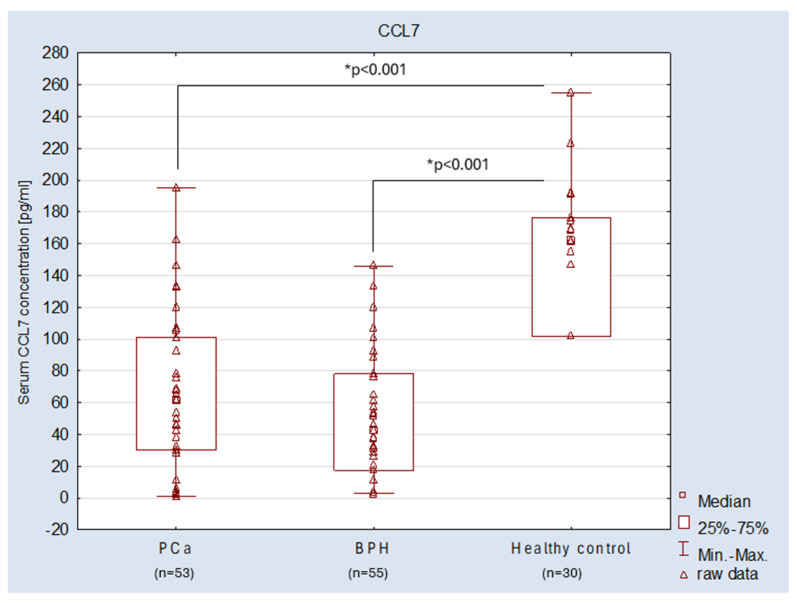
Serum concentrations of CCL7 in patients with prostate cancer (PCa), benign prostatic hyperplasia (BPH), and healthy controls. The box plots illustrate the median (central line), interquartile range (IQR; box), and minimum–maximum range (whiskers), while individual data points indicate single observations. Statistical analysis was performed using the Mann–Whitney U test.

**Figure 4:**
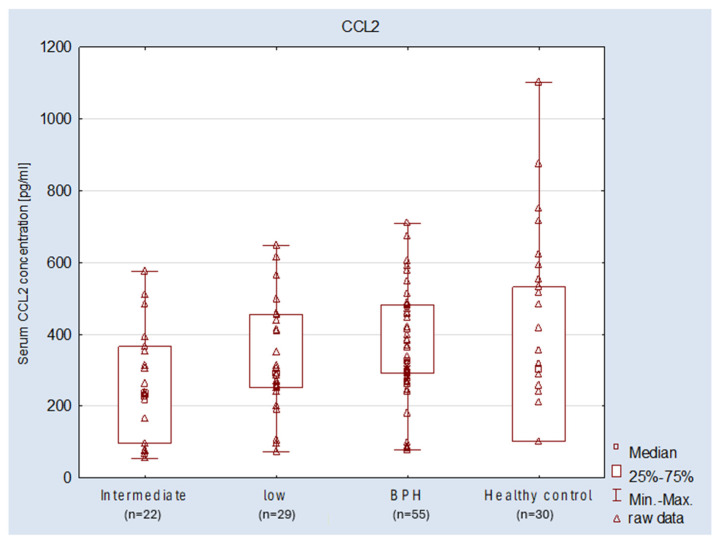
Serum concentrations of CCL2 in patients with low and intermediate risk of prostate cancer (PCa), BPH, and healthy controls. The box plots illustrate the median (central line), interquartile range (IQR; box), and minimum–maximum range (whiskers), while individual data points indicate single observations. Statistical analysis was performed using the Mann–Whitney U test.

**Figure 5:**
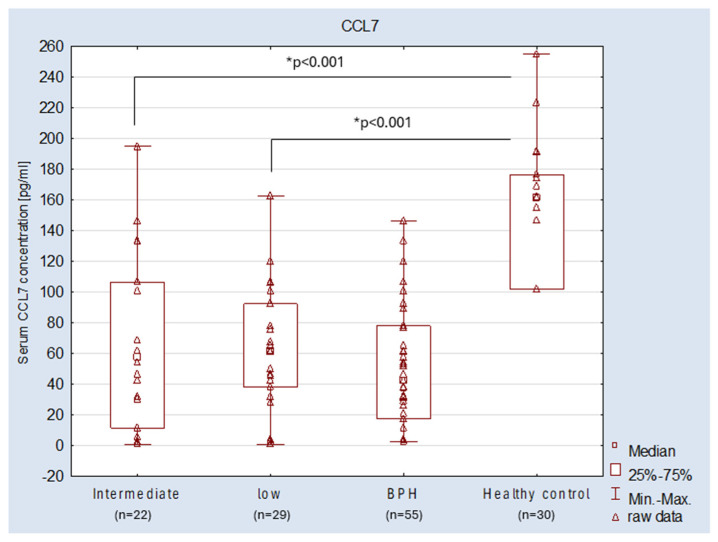
Serum concentrations of CCL7 in patients with low and intermediate risk of prostate cancer (PCa), BPH, and healthy controls. The box plots illustrate the median (central line), interquartile range (IQR; box), and minimum–maximum range (whiskers), while individual data points indicate single observations. Statistical analysis was performed using the Mann–Whitney U test.

The presence of both chemokines was confirmed in the serum of all study participants. The median CCL2 concentration was significantly lower in the PCa group (271.23 pg/mL) compared to BPH (325.27 pg/mL), while no significant differences were found between the other analyzed groups. In PCa patients, a progressive decrease in CCL2 chemokine concentration was observed with increasing PCa risk; however, there was no statistical significance between groups. The study demonstrated lower serum levels of CCL7 across all groups compared to CCL2. The concentration of the chemokine CCL7 in the serum of PCa patients was statistically significantly (*p* < 0.001) lower (61.45 pg/mL) compared to the control group (161.69 pg/mL). The lowest CCL7 concentration values were observed in the group of patients with BPH (42.63 pg/mL). However, no significant differences in the concentration of this chemokine were found between patients with PCa and BPH or between PCa subgroups with different clinical risks of tumor progression. These results suggest a potential association between serum CCL7 levels and the occurrence and risk of prostate cancer. The distributions of serum CCL2 and CCL7 concentrations in the studied groups are presented in Figure 2, Figure 3, Figure 4 and Figure 5, respectively.

Table 2 presents the diagnostic utility parameters of the chemokine CCL7 and tPSA. The chemokine CCL7 demonstrated a high sensitivity (SE) of 96.2%, with a specificity of 73.3%. Positive predictive value (PPV) and negative predictive value (NPV) were calculated, yielding 86.4% and 91.7%, respectively. Accuracy for this parameter was 88%, and the AUC reached 0.917. For comparison, tPSA demonstrated sensitivity, specificity, PPV, and NPV of 94.3%, 96.5%, 94.3%, and 96.5%, respectively, with an overall accuracy of 95.7% and an AUC of 0.993, indicating superior diagnostic performance in this cohort.

**Figure 6:**
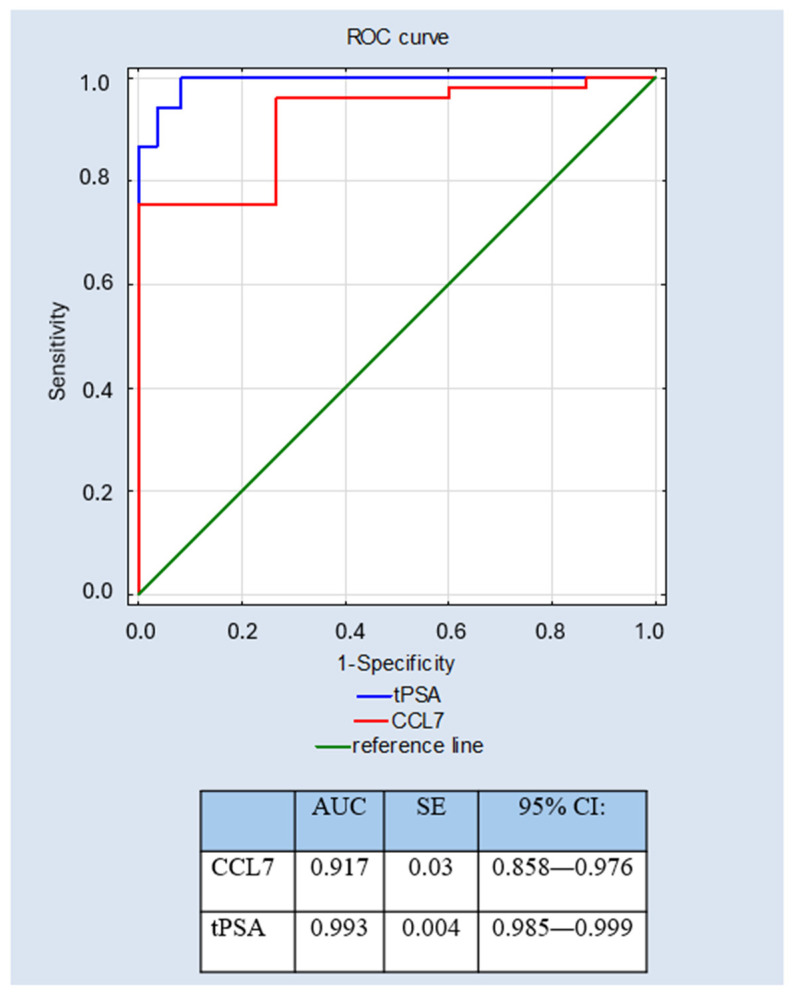
Receiver operating characteristic (ROC) curves for CCL7 and tPSA in the diagnosis of prostate cancer.

To assess diagnostic effectiveness, a receiver operating characteristic (ROC) curve analysis was performed for the tested CCL7 and tPSA, compared with a biomarker commonly used in prostate cancer diagnosis (Figure 6). After observing an association between decreased serum CCL7 levels and PCa, a receiver operating characteristic (ROC) curve analysis was performed using CCL7 as a destimulant variable. The results indicated that the area under the ROC curve (AUC) for CCL7 was 0.917 and was similar to the value obtained for tPSA (AUC = 0.993).

**Figure 7:**
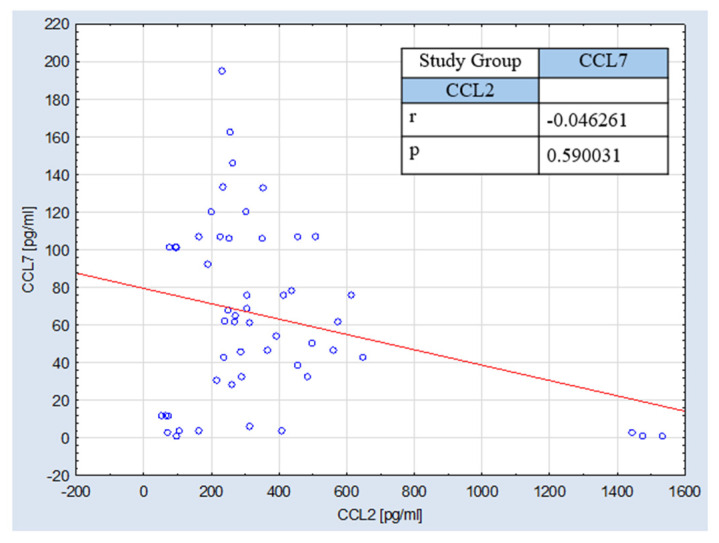
Spearman correlation between serum CCL2 and CCL7 concentrations in patients with prostate cancer.

To further investigate the strength and direction of monotonic associations between the analyzed variables, Spearman’s rank-order correlation coefficient was employed. Moreover, the relationship between CCL2 and CCL7 concentrations was visualized in a scatter plot, as presented in Figure 7. Spearman’s rank correlation analysis revealed no significant correlation between serum CCL2 and CCL7 concentrations in patients with PCa (r = −0.046, *p* = 0.590). These findings indicate that the serum levels of both chemokines were not associated and varied independently within the studied group of patients.

## 3. Discussion

Prostate cancer, the most common malignant neoplasm among men, with a steadily increasing incidence over the years, constitutes a global challenge for healthcare systems worldwide. Early and accurate diagnosis is crucial for improving the likelihood of detecting the disease at an early stage. Currently, diagnostic approaches are based on digital rectal examination, measurement of PSA, imaging of the prostate gland, and biopsy [16].

PSA remains the cornerstone of prostate cancer diagnosis. However, its limited specificity and inability to reliably distinguish between malignant and benign prostate lesions underscore the need to develop additional biomarkers that could complement current diagnostic strategies.

Scientific studies have demonstrated the diagnostic utility of PSA in identifying cancer at levels of 10 ng/dL or higher; however, challenges remain in detecting PCa at earlier stages of the disease [17,18,19]. Moreover, PSA demonstrates limited ability to distinguish indolent from aggressive disease, which may result in overinterpretation and the implementation of treatment not adequately matched to the clinical case [16,20]. Risk stratification of prostate cancer is based on PSA levels, histopathological grading according to the Gleason score (ISUP), and clinical staging using the TNM system, which allows classification into low-, intermediate-, and high-risk groups [21]. Despite its general diagnostic utility, PSA has certain limitations; therefore, there is ongoing interest in identifying non-invasive alternatives that could support and complement current diagnostic methods.

Increasing evidence points to a key role of chemokines in signaling pathways involved in carcinogenesis. These are chemotactic proteins belonging to the cytokine family, with molecular weights of 8–12 kDa, serving as a critical pathway for intercellular communication between tissue cells and components of the immune system [22]. Particular attention has been given to the CC-chemokine subfamily, which is associated with various biological and pathological processes in cancer, including chemotaxis, inflammation, cell migration, and immune responses to tumor-associated antigens [23]. In this study, we focused on the chemokines CCL2 and CCL7 by assessing their serum concentrations in patients diagnosed with PCa and stratified by PCa risk. The aim was to evaluate the correlation between their levels and disease progression. Additionally, we analyzed their concentrations in men with benign prostatic hyperplasia, using healthy volunteers as a reference group. For comparative purposes, tPSA, a routinely used parameter in prostate cancer diagnostics, was measured concurrently.

Particular interest in CCL2 and CCL7 stems from their involvement in the regulation of tumor–immune system interactions, which are increasingly recognized as key determinants of prostate cancer development and progression. Both chemokines belong to the monocyte chemoattractant protein (MCP) family and participate in the recruitment and activation of immune cells within the tumor microenvironment. Through modulation of leukocyte trafficking, inflammatory signaling, and stromal cell activity, they may contribute to the establishment of a microenvironment that either promotes or restrains tumor growth. Importantly, accumulating evidence suggests that alterations in chemokine signaling may occur not only locally within prostate tissue but also systemically, making circulating chemokines attractive candidates for minimally invasive biomarker research. Therefore, evaluating serum CCL2 and CCL7 concentrations may provide insight into the complex interplay between immune regulation, chronic inflammation, and prostate cancer biology [12,13].

The present analysis confirmed the presence of both chemokines in the serum of all study participants, which likely reflects that CCL2 and CCL7 are secreted by a wide variety of cell types under both physiological and pathological conditions. CCL2 is produced by numerous cell types, primarily monocytes and macrophages, but also epithelial cells, endothelial cells, fibroblasts, and cancer cells. CCL7 exhibits a similarly broad expression profile and may be synthesized by immune cells, stromal cells, epithelial cells, and tumor cells, indicating its significant role in regulating inflammatory and tumor microenvironments [24]. Importantly, within the tumor microenvironment, tumor-associated macrophages (TAMs) have recently been identified as a major source of both CCL2 and CCL7, and increased expression of these chemokines has been implicated in the establishment of an immunosuppressive microenvironment that supports tumor progression. These findings further support the biological relevance of circulating CCL2 and CCL7 as potential biomarkers reflecting tumor–immune interactions in cancer [12,25].

The range of serum CCL2 concentrations observed in our study was broader and higher compared to CCL7 across all groups. This suggests potentially different secretion kinetics of these chemokines and a more dynamic inflammatory response of CCL2 compared to CCL7 [26,27,28]. Moreover, these differences may result from the fact that CCL2 is the most potent ligand activating CCR2 signaling [29,30]. Binding of the ligand to CCR2 activates a G protein-coupled receptor signaling pathway, initiating intracellular cascades involving PI3K/AKT, MAPK/p38, protein kinase C (PKC), calmodulin-dependent protein kinase II, and JAK/STAT3 pathways. These processes lead to cytoskeletal reorganization and phospholipase C-dependent calcium release, resulting in inhibition of apoptosis and promotion of angiogenesis [31,32,33]. It has also been reported that the CCL2–CCR2 interaction increases the production of integrin αvβ3, enhancing invasion and migration of PCa cells [34].

Our study demonstrated that serum CCL2 levels were significantly lower in patients with PCa (271.23 pg/mL) compared to men with BPH (325.27 pg/mL). These differences may reflect distinct biological processes underlying these conditions despite their shared anatomical location. BPH is characterized by a predominance of active inflammatory responses with infiltration of T lymphocytes and M1 macrophages and elevated levels of pro-inflammatory cytokines. In contrast, PCa exhibits an immunosuppressive tumor microenvironment, with dominance of Treg, M2 macrophages, and increased expression of immune checkpoint molecules such as PD-L1, enabling tumor cells to evade immune responses [35,36].

The observed decrease in circulating CCL2 concentrations in PCa may appear unexpected, as numerous experimental studies have demonstrated increased activity of the CCL2/CCR2 axis within prostate tumor tissue. However, tissue expression and serum concentrations do not necessarily reflect the same biological processes. CCL2 may be produced and retained locally within the tumor microenvironment, where it promotes recruitment of tumor-associated macrophages, angiogenesis, and tumor cell migration through paracrine signaling. Consequently, enhanced local utilization of CCL2 may not be accompanied by elevated systemic concentrations. Moreover, chronic inflammation associated with BPH may stimulate greater systemic production and release of CCL2 than the more immunosuppressive microenvironment observed in prostate cancer. Therefore, the lower serum concentrations observed in our PCa group may reflect differences between local chemokine activity and sequestration in the tumor microenvironment and circulating chemokine profiles rather than diminished biological importance of the CCL2 pathway itself [37].

No significant differences in CCL2 levels were observed between the other analyzed groups. Marsland M. et al. also evaluated serum CCL2 levels in prostate cancer patients and additionally assessed tissue expression of CCL2 and CCR2. Their findings differed from ours, as they observed significantly higher serum CCL2 levels in PCa patients compared to BPH and healthy controls. However, similarly to our results, they found no significant differences between tumors of varying grades. Furthermore, analysis of CCL2 expression in prostate cancer biopsies revealed no correlation with clinicopathological parameters, disease progression, or treatment outcomes [38]. Iwamoto H. et al. demonstrated the clinical relevance of serum CCL2 by showing a correlation between higher levels and poorer survival [39], and in a separate long-term study confirmed its prognostic value in PCa patients [40]. Elevated CCL2 levels have also been reported in other malignancies, including colorectal cancer [41] and malignant pleural mesothelioma [42]. Discrepancies between studies may be attributed to heterogeneity in patient populations, including differences in disease stage, inflammatory response, and tumor microenvironment characteristics. Taken together, the available evidence suggests that circulating CCL2 reflects complex interactions between inflammation and tumor biology rather than prostate cancer-specific processes.

Our study also showed that serum CCL7 levels were significantly lower in patients with prostate cancer compared to healthy controls. One possible explanation is local accumulation of CCL7 within tumor tissue; however, tissue expression was not evaluated in the present study. CCL7 exhibits broad receptor specificity, acting as a ligand for CCR1, CCR2, CCR3, and CCR5, which facilitates its uptake and utilization in tissues, including the tumor microenvironment [43]. Higher levels observed in healthy individuals may reflect physiological immune activity, whereas prostate cancer is associated with modulation of the chemokine axis and altered inflammatory responses [44,45]. The lowest CCL7 levels were observed in patients with BPH. No significant differences were found between PCa and BPH patients or among PCa subgroups with different clinical risk levels. These findings suggest a potential role for CCL7 in distinguishing healthy individuals from prostate cancer patients; however, its utility as a predictor of disease progression or treatment outcomes appears limited. Literature reports indicate that serum CCL7 levels in cancer patients may either increase or decrease depending on tumor type. Karan D. et al. evaluated 40 chemokines and cytokines in the serum of prostate cancer patients and healthy controls of both African American and Caucasian origin and similarly reported significantly lower CCL7 levels in cancer patients regardless of race [46]. Interestingly, the direction of changes in circulating CCL7 appears to be tumor-dependent. In contrast to our findings and those reported by Karan et al. in prostate cancer, Chidimatsu et al. demonstrated that patients with metastatic colorectal cancer exhibited elevated pre-treatment serum CCL7 concentrations, which were independently associated with poorer overall survival and improved prognostic performance when combined with carcinoembryonic antigen (CEA). These findings suggest that circulating CCL7 may reflect distinct biological processes across malignancies and that its clinical significance depends on tumor-specific regulation of chemokine production, release, and utilization within the tumor microenvironment [47]. Conversely, Bai Y. et al. demonstrated significantly reduced expression of both CCL2 and CCL7 in prostate tumor tissue compared to BPH tissue [48]. In our previous study on clear cell renal cell carcinoma (ccRCC), we also observed decreased serum CCL7 levels in patients compared to healthy controls [49]. Most studies have focused on CCL7 expression in tumor tissues, including colorectal metastases [50], ovarian cancer [51], and non-small cell lung cancer [52], where increased expression has been associated with disease progression. Collectively, these observations indicate that serum CCL7 cannot be regarded as a universal cancer biomarker. Rather, its circulating concentration appears to be strongly influenced by tumor type and the biological characteristics of the tumor microenvironment, emphasizing the need for cancer-specific interpretation of CCL7 as a diagnostic or prognostic marker.

Our study results indicate that CCL7 may have diagnostic potential primarily as a marker for distinguishing patients with prostate cancer from healthy individuals; however, this chemokine’s limited ability to differentiate prostate cancer from BPH significantly limits its current clinical utility. Given that distinguishing between these two conditions is one of the main challenges in the diagnosis of prostate cancer, CCL7 should not be considered a standalone diagnostic marker at this stage. This is supported by a high area under the ROC curve (AUC = 0.917), comparable to that of tPSA (AUC = 0.993), and high sensitivity (96.2%). However, moderate specificity (73.3%) suggests a risk of false-positive results, limiting its use as a standalone diagnostic marker. High predictive values, particularly an NPV of 91.7%, indicate potential utility in ruling out disease. Interestingly, CCL7 demonstrated better discriminatory performance than CCL2 despite being substantially less studied in prostate cancer. Given its ability to interact with multiple chemokine receptors, circulating CCL7 may reflect broader alterations in tumor–immune communication than individual chemokine pathways. Nevertheless, the lack of significant differences between PCa and BPH limits its clinical applicability as an independent biomarker. Notably, there is a lack of studies directly assessing the diagnostic value of serum CCL7 using ROC analysis, preventing direct comparison and highlighting the need for further research.

It should also be emphasized that serum chemokine profiles reflect a complex interplay of multiple physiological and pathological processes, which may reduce their specificity for PCa due to non-cancer-related factors.

CCL2 and CCL7 are pleiotropic inflammatory chemokines involved in a wide range of chronic inflammatory processes. Consequently, their circulating levels may be increased in individuals with obesity [53,54]. Moreover, metabolic disorders, including insulin resistance and type 2 diabetes mellitus [55], as well as cardiovascular diseases such as ischemia, reperfusion injury, fibrotic heart failure [56,57], and hypertension [58], may influence serum concentrations of these chemokines and should therefore be considered potential confounding factors. Several commonly prescribed medications, particularly statins, metformin, angiotensin-converting enzyme inhibitors, and angiotensin receptor blockers, have been shown to modulate inflammatory signaling pathways involving CCL2. Therefore, concomitant pharmacotherapy may represent a potential confounding factor when interpreting circulating CCL2 concentrations. Evidence for circulating CCL7 is currently much more limited, although similar regulatory mechanisms have been proposed [59,60,61]. These variables constitute important determinants that can mask or modify changes associated with carcinogenesis. Therefore, clinical evaluation requires careful interpretation and consideration of the full clinical context as a potential source of confounding variables.

Our findings suggest distinct patterns of circulating CCL2 and CCL7 expression in prostate cancer. While CCL2 showed limited diagnostic utility, its potential biological relevance in prostate cancer should not be excluded, given its established role in tumor–immune interactions. CCL7 demonstrated favorable diagnostic performance mainly in distinguishing prostate cancer patients from healthy individuals and may be a promising component of future multimarker strategies to improve PCa detection. However, the lack of significant differences between PCa and BPH limits its current clinical applicability, as distinguishing these conditions remains one of the major challenges in prostate cancer diagnostics. PSA remains superior in diagnostic accuracy; therefore, CCL7 should be considered only as a supplementary biomarker. Importantly, the limited number of patients representing higher-risk disease prevents definitive conclusions regarding the potential role of CCL7 as a marker of disease aggressiveness or progression.

### Limitations and Future Perspectives

Despite the identification of key findings, it is important to acknowledge that the present study is subject to several limitations. The relatively small sample size, particularly within the group of patients with intermediate-risk prostate cancer, as well as the very limited representation of high-risk cases (*n* = 2), may have reduced the statistical power and the ability to detect subtle differences between clinical stages. Furthermore, the single-center design of the study may restrict the generalizability of the findings to broader populations. Accordingly, the results should be interpreted with caution and regarded as preliminary. Although the control group consisted of healthy volunteers with no history of malignancy, autoimmune disorders, chronic inflammatory conditions, or active infections, circulating chemokine levels may still be influenced by other unmeasured biological or environmental factors. Therefore, the presence of additional confounding variables cannot be entirely excluded. Moreover, the concentrations of chemokines CCL2 and CCL7 were assessed exclusively in serum, without parallel validation at the tissue level, which limits the ability to draw definitive conclusions regarding the relationship between circulating chemokine levels and tumor microenvironment activity. The integration of serum-based and tissue-based analyses in future studies would enable a more comprehensive understanding of the biological role of these chemokines in PCa progression. Finally, larger-scale, multicenter studies are warranted to validate the observed associations and to determine the potential clinical utility of CCL2 and CCL7 as diagnostic or prognostic biomarkers.

## 4. Materials and Methods

The study included 53 patients diagnosed with PCa, who underwent prostatectomy at the Department of Urology, Independent Public Health Care Center of the Ministry of the Interior and Administration in Białystok, Poland.

According to the European Association of Urology (EAU) classification (2025) (Table 3), PCa patients were divided into three groups: 29 individuals with low risk, 22 with intermediate risk, and 2 with high risk of disease progression. The high-risk group was included only in the overall PCa group and was not analyzed as a separate subgroup due to insufficient sample size. Detailed demographic and clinical characteristics of the study population are presented in Table 4 and Table 5.

In addition, a comparative group of 55 men diagnosed with benign prostatic hyperplasia (BPH) (age range: 48–85 years) was included. Individuals with a history of cancer treatment, active infectious diseases, autoimmune disorders, chronic inflammatory conditions, or current immunomodulatory therapy were excluded from participation. The control group consisted of 30 healthy volunteers (mean age 58 years, range 51–67 years) without a history of malignancy, autoimmune disorders, chronic inflammatory diseases, or current infection. Baseline demographic and clinical characteristics, including age and PSA levels, were compared between study groups to identify potential differences that could represent confounding factors affecting the interpretation of chemokine concentrations (Table 4 and Table 5).

Biological material consisted of blood serum collected from both study participants and controls before the initiation of any therapeutic intervention, in order to eliminate potential pharmacological effects on chemokine levels. Furthermore, none of the enrolled subjects reported long-term use of medications known to influence immune system function. Peripheral blood was collected from each participant in a tube without an anticoagulant. After clot formation, the material was centrifuged to isolate the serum, separated, and stored at −80 °C until analysis. The chemokines studied were assayed and measured using a Luminex 200 analyzer and Luminex Human Discovery assay plates, supplied by R&D Systems, Abingdon, UK. This bead-based multiplex immunoassay technology allows the simultaneous quantification of multiple analytes within a single sample through fluorescence-based detection. All standards, controls, and samples were analyzed in duplicate in accordance with the manufacturer’s protocols. Quantitative analysis based on mean fluorescence intensity (MFI) was performed using xPONENT 3.1.971.0 software (Luminex, Austin, TX, USA). The clinical diagnosis of patients was established based on histopathological examination of material obtained during prostatectomy.

The research protocol was approved by the Medical University of Białystok’s Human Care Committee located in Białystok, Poland (Approval Nr APK.002.482.2025). All patients from the tested and control groups gave their informed consent for the examination.

Statistical analyses were conducted using Statistica 13.0 software. The Shapiro–Wilk test indicated that the data did not follow a normal distribution. Therefore, nonparametric tests, including the Mann–Whitney U test and the Kruskal–Wallis test, were applied. Post hoc pairwise comparisons were performed when appropriate. Continuous variables are presented as mean ± standard deviation (SD) or median with minimum–maximum range, depending on data distribution. Diagnostic performance indicators: sensitivity, specificity, positive predictive value, and negative predictive value were calculated based on optimal cutoff values determined using the Youden index. Receiver operating characteristic curves were constructed for chemokines CCL2 and CCL7, and Spearman’s rank correlation analysis was performed to evaluate associations between variables. A *p*-value < 0.05 was considered statistically significant.

## 5. Conclusions

This study extends the current evidence by directly comparing the diagnostic utility of the well-characterized CCL2 with the less extensively investigated CCL7 in the same cohort of patients with prostate cancer, benign prostatic hyperplasia, and healthy controls. Among the analyzed chemokines, CCL7 demonstrated promising but preliminary diagnostic utility, whereas the clinical relevance of circulating CCL2 remains uncertain. Serum CCL7 concentrations were significantly lower in patients with prostate cancer and demonstrated favorable diagnostic performance in distinguishing prostate cancer patients from healthy controls. In contrast, CCL2 exhibited limited diagnostic utility and poor discriminatory capacity. These findings suggest that CCL7 may serve as a complementary inflammatory marker in multimarker diagnostic panels for prostate cancer. However, the small sample size and limited representation of high-risk patients restrict the interpretation of CCL7 in relation to disease progression and risk stratification. Further studies are needed to validate these findings and confirm the diagnostic value of CCL7 in prostate cancer.

## Figures and Tables

**Table 1:** Serum concentrations of chemokines CCL2, CCL7, and tPSA in cancer patients and controls.

Group	Mean	Median	Min.	Max.	SD
CCL2					
PCa Group	355.87	271.23	54.10	1534.38	316.54
PCa Low Risk	400.37	289.06	72.73	1534.38	340.44
PCa Intermediate Risk	308.37	235.45	54.10	1443.44	294.24
BPH Group	458.10	325.27	77.70	1788.27	369.45
Control Group	369.77	302.72	101.00	1103.22	259.13
*p* ^a^ = 0.40; *p* ^b^ = 0.03; *p* ^c^ = 0.68; *p* ^d^ = 1.00; *p* ^e^ = 1.00; *p* ^f^ = 1.00; *p* ^g^ = 0.051					
CCL7					
PCa Group	65.02	61.45	0.95	194.98	47.29
PCa Low Risk	62.53	61.99	0.95	162.50	40.29
PCa Intermediate Risk	68.60	57.71	0.95	194.98	55.02
BPH Group	52.05	42.63	2.84	146.09	36.99
Control Group	159.50	161.69	102.00	254.89	44.40
*p* ^a^ < 0.001; *p* ^b^ = 0.62; *p* ^c^ < 0.001; *p* ^d^ < 0.001; *p* ^e^ = 1; *p* ^f^ < 0.001; *p* ^g^ = 1.00					
tPSA					
PCa Group	9.13	9.00	4.90	13.00	2.11
PCa Low Risk	9.23	9.00	4.90	13.00	2.24
PCa Intermediate Risk	9.06	8.95	6.50	13.00	2.08
BPH Group	3.20	3.10	0.91	7.10	1.62
Control Group	3.18	2.9	1.7	4.7	0.79
*p* ^a^ < 0.001; *p* ^b^ < 0.001; *p* ^c^ = 1.00; *p* ^d^ < 0.001; *p* ^e^ < 0.001; *p* ^f^ < 0.001; *p* ^g^ < 0.001					

SD: Standard deviation; Statistically significant differences were defined as comparisons resulting in *p* < 0.05. Data are expressed as pg/mL; *p* ^a^—cancer patients vs. control; *p* ^b^—cancer vs. BPH; *p* ^c^—control vs. BPH; *p* ^d^—low risk vs. control; *p* ^e^—low risk vs. BPH; *p* ^f^—intermediate risk vs. control; *p* ^g^—intermediate risk vs. BPH.

**Table 2:** Diagnostic performance parameters of serum CCL2 and CCL7 in prostate cancer.

	Cut-Off	Diagnostic Sensitivity (%)	Diagnostic Specificity (%)	Positive Predictive Value (%)	Negative Predictive Value (%)	Accuracy (%)	AUC
CCL7	146.09	96.2	73.3	86.4	91.7	88.0	0.917
tPSA	6.5	94.3	96.5	94.3	96.5	95.7	0.993

Cut-off values were established using the Youden index to optimize the combined sensitivity and specificity. CCL7 was considered a destimulant due to its inverse association with PCa, with lower serum concentrations observed in patients with PCa.

**Table 3:** European Association of Urology risk groups for biochemical recurrence of prostate cancer [52].

Risk Group		Criteria	Clinical Stage
Low-risk		ISUP grade 1 and PSA < 10 ng/mLand cT1-2a *	Localised
Intermediate-risk	Favourable	ISUP grade 2 and PSA < 10 ng/mL and cT1-2b * Or ISUP grade 1 and PSA 10–20 ng/mL and cT1-2b * Or ISUP grade 1 and PSA < 10 ng/mL and cT2b *	
	Unfavourable	ISUP grade 2 and PSA 10–20 ng/mL and cT1-2b * Or ISUP grade 3 and cT1-2b *	
High-risk		ISUP grade 4/5 Or PSA > 20 ng/mL Or cT2c *	
		cT3-4 * and/or cN+ ** any ISUP grade * any PSA	Locally advanced

Abbreviations: ISUP—International Society for Urological Pathology; PSA—prostate-specific antigen. * Based on digital rectal examination. ** Based on CT and/or bone scan.

**Table 4:** Clinical characteristics of the control group and patients with prostate cancer (PCa).

Characteristic	Control Group (*n* = 30)	BPH Group (*n* = 29)	PCa Group (*n* = 51)	*p*-Value
Age (years), mean ± SD	58	72.3 ± 5.8	68.6 ± 6.3	*p* ^a^ < 0.001
				*p* ^b^ < 0.001
				*p* ^c^ = 0.010
Age range	51–67	60–85	50–88	–
PSA (ng/mL), mean ± SD	3.18 ± 0.79	3.20 ± 1.62	9.13 ± 2.11	*p* ^a^ < 0.001
				*p* ^b^ < 0.001
				*p* ^c^ = 0.001
PSA range (ng/mL)	1.7–4.7	0.91–7.10	4.9–13.0	–
Clinical stage, *n* (%)				
Localized	–	–	50 (98.0)	–
Locally advanced	–	–	1 (2.0)	–
EAU risk group, *n* (%)				
Low risk	–	–	26 (51.0)	–
Intermediate risk	–	–	23 (45.1)	–
High risk	–	–	2 (3.9)	–

Post hoc comparisons: Control vs BPH, Control vs PCa, and BPH vs PCa; *p* ^a^—cancer patients vs. control; *p* ^b^—cancer vs. BPH; *p* ^c^—control vs. BPH.

**Table 5:** Clinical characteristics of PCa patients according to EAU risk classification.

Characteristic	Low Risk (*n* = 26)	Intermediate Risk (*n* = 23)	High Risk (*n* = 2)	*p*-Value (Low vs. Intermediate)
Age (years), mean ± SD	68.4 ± 7.2	68.8 ± 5.2	73.0 ± 2.8	0.83
Age range	50–88	59–76	71–75	-
PSA (ng/mL), mean ± SD	9.25 ± 2.63	8.68 ± 2.05	9.40 ± 0.99	1.00
PSA range (ng/mL)	4.9–14.0	3.5–15.0	8.7–10.1	-
Clinical stage, *n* (%)				
Localized	26 (100)	22 (95.7)	1 (50.0)	-
Locally advanced	0	1 (4.3)	1 (50.0)	-
Gleason score, *n* (%)				
GS 6	25 (96.2)	0	0	-
GS 7	1 (3.8)	23 (100)	0	-
GS ≥ 8	0	0	2 (100)	-

## Data Availability

The original contributions presented in this study are included in the article. Further inquiries can be directed to the corresponding author.
